# Ferromagnetic Interlayer Exchange Coupling in a Few Layers of CrSBr on a Gold Thin Film

**DOI:** 10.1021/acs.nanolett.6c01730

**Published:** 2026-08-03

**Authors:** Rixt Bosma, Darius A. Pacurar, Daniel Sade, Jingbo Wang, Nicholas Dale, Cameron W. Johnson, Sergii Grytsiuk, Alexander Rudenko, Alexander Stibor, Malte Rösner, Marcos H. D. Guimarães, Roberto Lo Conte

**Affiliations:** † Zernike Institute for Advanced Materials, 3647 University of Groningen, Groningen 9747 AG, The Netherlands; ‡ 1666 Molecular Foundry, Lawrence Berkeley National Laboratory, Berkeley, California 94720, United States; ¶ Institute for Molecules and Materials, Radboud University, Nijmegen 6525 AJ, Netherlands; § Materials Science Division, Lawrence Berkeley National Laboratory, Berkeley, California 94720, United States; ∥ Faculty of Physics, Bielefeld University, Bielefeld 33615, Germany

**Keywords:** 2D magnets, van der Waals materials, interface effects, spin-polarized low energy electron microscopy.

## Abstract

The two-dimensional character of van der Waals magnets allows for efficient control of their properties via proximity effects and electrical stimuli, making them promising candidates for application in spin electronics. We use spin-polarized low-energy electron microscopy to directly image the magnetic texture of thin CrSBr on top of a Au film, discovering a ferromagnetic ground state. We argue that the stabilization of the ferromagnetic ordering—as compared to the conventional antiferromagnetic one—is obtained via electron transfer from the Au film to the CrSBr flakes, in agreement with ab initio density functional theory calculations. Reflected-electron spectroscopy shows clear differences in the unoccupied density of states between a few layers of CrSBr on Au and bulk CrSBr, pointing toward electronic band structure modification in thin CrSBr. This work sheds light on the possibility of tuning the magnetic properties of two-dimensional magnets via substrate engineering.

## Graphical abstract

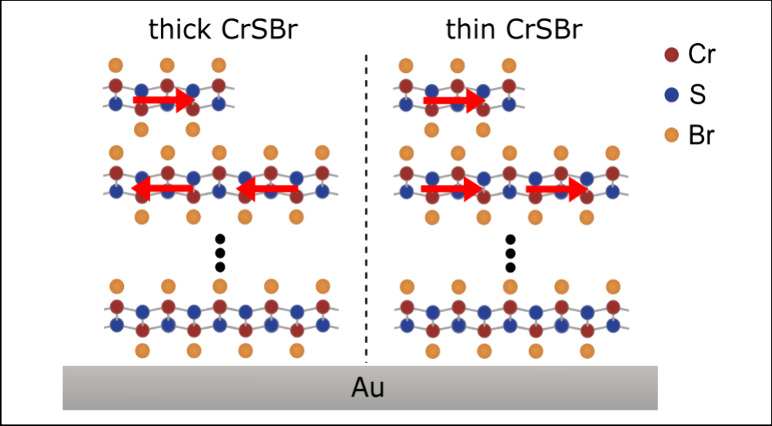

Two-dimensional (2D) van der Waals (vdW) magnetic materials are a promising platform for the exploration of magnetism in low dimensions.^1,2^ Because of their reduced dimensionality, 2D vdW magnets allow for the efficient tuning of their magnetic properties through external stimuli. As a result, these materials hold high promise for applications such as magnetic data storage and logic devices.

Although vdW magnetic materials and the tuning of their properties through electrostatic gating have been studied extensively from an experimental point of view,^3–7^ the impact of the substrate on their magnetic properties has been much less explored experimentally, despite several theoretical predictions.^8–11^ For applications in actual devices, this is of particular importance since the substrate and electrical contacts could locally influence the properties of the system, resulting in magnetic ground states which differ from what is expected for pristine and free-standing 2D magnets. Additionally, these interactions could be used to tune material properties,^9,12^ leading to local control of magnetism through substrate engineering.

The prototypical vdW magnet CrSBr represents a promising candidate for spintronic applications. CrSBr is particularly interesting due to its decent air stability and relatively high magnetic ordering temperature of 132 K.^13^ It was shown that the magnetic ordering of CrSBr can be tuned from the A-type antiferromagnetic ordering to the ferromagnetic ordering via ions intercalation^14–16^ or electric gating,^5^ due to a modification of the interlayer magnetic exchange. Previous studies based on band structure mapping by angular-resolved photoelectron spectroscopy (ARPES) offered an initial understanding of how the electronic and magnetic properties of CrSBr are modified by the proximity to metallic substrates.^9,12,17^ Furthermore, the imaging of the magnetic ground state of thin CrSBr flakes on substrates has also been performed, mainly via nitrogen-vacancy (NV) center microscopy,^18,19^ magnetic force microscopy,^20^ and SQUID on tip microscopy.^21^ However, as of today, direct imaging of the modification of the magnetic ground state of few-layer CrSBr flakes via interface effects is still missing.

Here, we report on the magnetic imaging of few-layer CrSBr flakes via spin-polarized low-energy electron microscopy (SPLEEM). We discovered a ferromagnetic ground state in few monolayer-thick CrSBr flakes in direct proximity to a metallic gold (Au) film on a SiO_*x*_/Si substrate. Furthermore, a modification of the electronic band structure of the thin CrSBr flake in proximity to the Au film was detected via spatially resolved reflected electron spectroscopy (RES). The emergence of a ferromagnetic interlayer exchange coupling in CrSBr was analyzed through first-principles density functional theory calculations, which suggest charge transfer at the CrSBr-Au interface as a likely explanation for the observed ferromagnetic ground state.

CrSBr crystallizes into an orthorhombic structure that comprises double layers of chromium sulfide, capped on both sides by an anionic bromide layer. Monolayers of CrSBr are held together by weak vdW forces. While the isotropic exchange interactions between neighboring Cr atoms within each layer are ferromagnetic (FM), the exchange coupling between adjacent layers is antiferromagnetic (AFM), resulting in an A-type antiferromagnetic ground state in pristine CrSBr.^22^ Furthermore, its orthorhombic crystal structure gives rise to pronounced uniaxial magnetic anisotropy, with the easy axis aligned along the [010] direction,^10^ defined as the *b*-direction in the following discussion.

A schematic of the samples prepared for this study is shown in Figure 1a. Thin flakes of CrSBr are transferred onto a Au­(20 nm)/Ti­(5 nm)/SiO_*x*_/Si substrate via exfoliation in a glovebox under inert nitrogen atmosphere. The sample was degassed at ca. 200 °C (well below the decomposition temperature of CrSBr of 800–850 °C^23^) for 15 h in ultrahigh vacuum ( $∼10^{-10}$ mbar) conditions before imaging, in order to remove potential surface contamination deriving from the stacking process and/or the exposure to air during the transfer from the glovebox to the microscope. Figure 1b reports a low-energy electron microscopy (LEEM) image of one of the prepared CrSBr flakes on Au/SiO_*x*_/Si substrate. The inset shows a low-energy electron diffraction (LEED) pattern acquired from the very same CrSBr flake, which allows us to confirm the main in-plane crystallographic directions of the CrSBr flake. The LEEM image shows steps in between different atomic terraces present at the CrSBr surface. The topography of the CrSBr surface is further characterized via atomic force microscopy (AFM) and shown in Figure 1c, offering a more accurate visualization of multiple terraces with different thicknesses.

**Figure 1:**
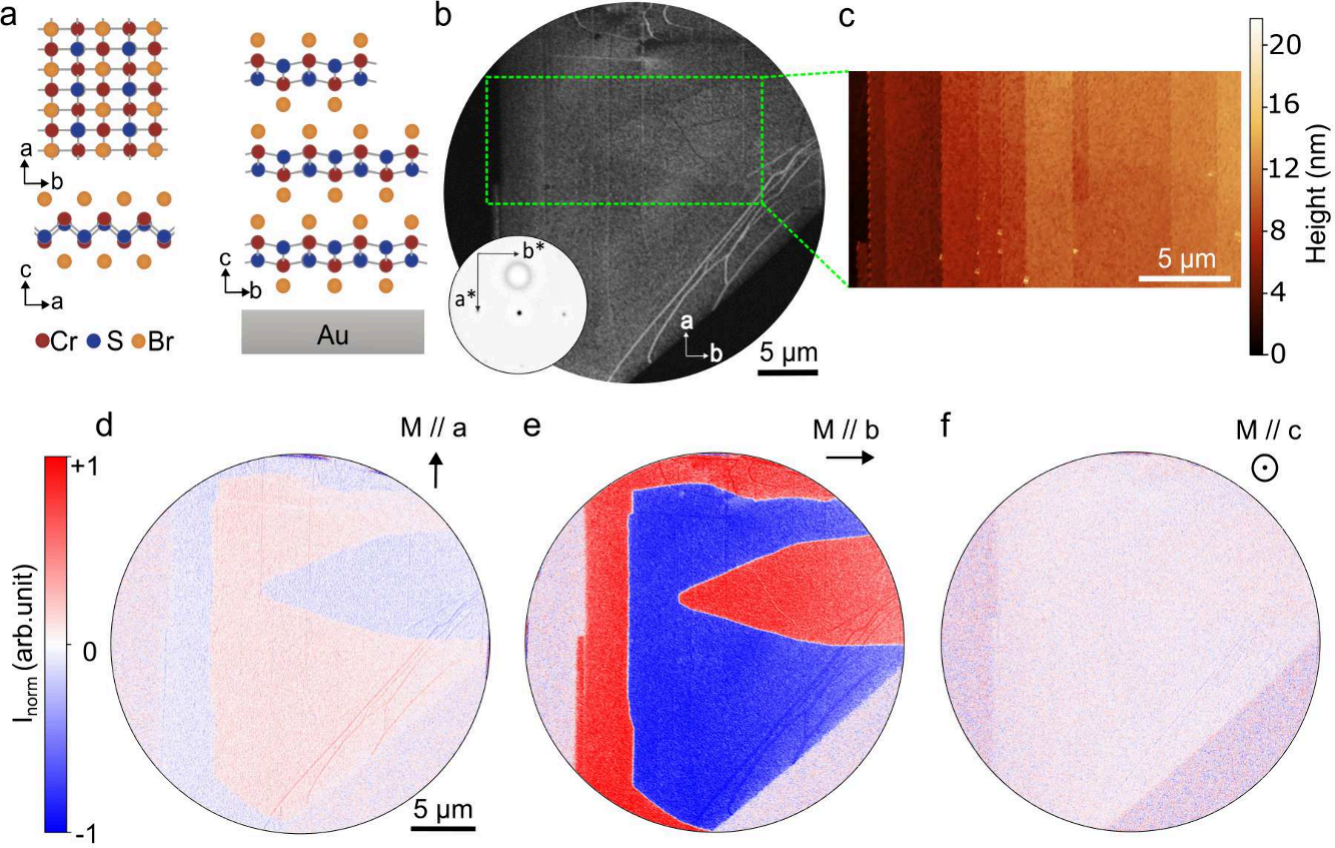
Schematic of the prepared CrSBr-flake/Au (20 nm)-thin film sample and its structural and magnetic characterization. **a** The CrSBr flake has an orthorhombic crystal structure. The material is placed on a polycrystalline Au film. **b** Low-energy electron microscopy (LEEM) image of the investigated flake. The central area of this flake is imaged with atomic force microscopy (AFM) and displayed in **c**. The inset shows the measured low-energy electron diffraction pattern at a bias voltage of 31.6 V. **d–f** Spin-polarized LEEM (SPLEEM) images of the CrSBr flake with spin polarization along the three main crystallographic directions, acquired with a bias voltage of 7.5 V. **d**, **e** SPLEEM images of the same CrSBr flake in **b** taken with spin polarization approximately along the *a*- and *b*-axis, respectively. The SPLEEM contrast is maximal in **e**, consistent with a magnetic easy axis parallel to the *b*-axis. **f** SPLEEM image for spin polarization along the *c*-axis, showing no magnetic contrast.

The magnetic ground state in the CrSBr flake is characterized via low-temperature spin-polarized LEEM (SPLEEM).^24^ The SPLEEM images were acquired after cooling the sample well below the ordering temperature of the CrSBr flake via a liquid-He flow cryostat (actual temperature at the sample is ca. 30 K). The acquired low-temperature SPLEEM images are shown in Figure 1d–f, which reveal the magnetic contrast along the three main crystallographic directions of the CrSBr flake. The high magnetic contrast in Figure 1e confirms a magnetic easy axis along the in-plane *b* axis. The weak magnetic contrast in Figure 1d is understood as a small misalignment between the electron beam spin polarization and the *a* axis of the CrSBr flake. As LEEM is strongly surface sensitive, the imaged contrast primarily corresponds to the top CrSBr layer. The SPLEEM images reveal the presence of a magnetic multidomain pattern, which does not follow the terrace pattern observed via LEEM (Figure 1b) and AFM (Figure 1c), as expected for an A-type antiferromagnet. Additional evidence of such a phenomenon was obtained also for other CrSBr flakes on a different Au thin film, as discussed in Supporting Information Section S3. These observations clearly point toward a different magnetic ground state hosted by the CrSBr flake, whose nature is discussed in more detail in the following.

Figure 2 presents a direct comparison between the magnetic domains present in the CrSBr flake and the topography of the magnetic flake. The SPLEEM image in Figure 2a shows the observed magnetic domain pattern of the CrSBr flake, with distinct red and blue regions indicating magnetic domains with opposite magnetization. The magnetic domain walls are visible as sharp transition regions between the domains. The dashed black line highlights the line-cut along which the magnetic contrast and the sample’s topography are directly compared.

**Figure 2:**
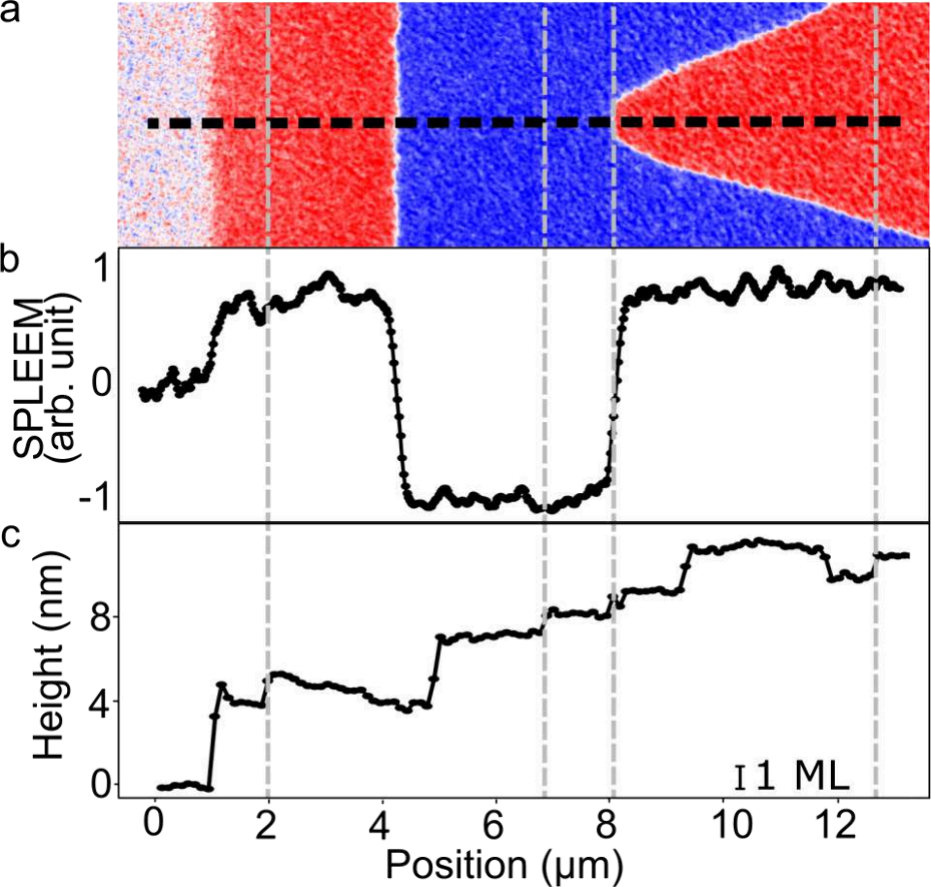
Comparison between observed magnetic contrast and CrSBr thickness. **a** Zoom-in of the SPLEEM image in Figure 1e, indicating the line along which the cut is taken. **b** Magnetic contrast along the line displayed in panel **a**. The contrast is averaged over 10 pixels. **c** The topographic profiles along the same lines, obtained from the AFM image in Figure 1c. Several of the monolayer steps present in the CrSBr flake, indicated by the vertical gray dotted lines, are found to be located in the middle of ferromagnetic domains.

Figure 2b shows the magnetic contrast along the dashed black line indicated in Figure 2a, with a positive/negative magnetic contrast for red/blue magnetic domains, respectively. Finally, Figure 2c shows the CrSBr flake’s topography along the same line. This allows for the direct comparison between the observed magnetic contrast and the flake’s topography. Importantly, single monolayer (1 ML ≈ 0.8 nm)^25^ steps present at the CrSBr surface—indicated in Figure 2c by vertical gray dotted lines—do not correspond to a change in the magnetic contrast shown in Figure 2b. While an inversion of the magnetic contrast is observed at the 1 ML-step in proximity to the apex of the cone-shaped domain, 1 ML-steps elsewhere do not correspond to a magnetization inversion. This observation is directly in conflict with an A-type antiferromagnetic ground state expected for free-standing CrSBr flakes and demonstrates the emergence of a ferromagnetic interlayer coupling in the studied CrSBr/Au system.

To verify that the ferromagnetic ground state reported in Figure 2 is not a one-time *freezing* of the particular magnetic domain pattern due to imperfections in the CrSBr flake but rather a general property of the system under investigation, we performed several thermal cycles where we repeatedly warmed up the sample above its ordering temperature, *T*_C_ ∼ 132 K and subsequently cooled it down below *T*_C_ and observed the emerging domain pattern after each cycle. The results of this experiment are shown in Figure 3. In Figure 3a there is an optical microscopy image of the same CrSBr flake discussed in Figure 1. Lighter/darker contrast indicates thinner/thicker parts of the 2D magnet. The green line indicates the position of a topographic step. Figure 3b shows the initial magnetic domain pattern in the entire CrSBr flake (see top panel). Figure 3c–f shows the magnetic domain pattern that formed after each warming-cooling cycle. Each SPLEEM image in the top row of Figure 3 shows the presence of a magnetic domain pattern which is consistent with the presence of a ferromagnetic order, where ferromagnetic domains are separated by domain walls which do not generally coincide with the 1 ML steps present in the CrSBr flake (as discussed in detail in Figure 2). On the contrary, we observe magnetic domains with the same magnetization direction which go across atomic steps of the CrSBr flake. This provides additional evidence for the emergence of a ferromagnetic interlayer exchange in the thin CrSBr flake in direct proximity to the metallic Au film.

**Figure 3:**
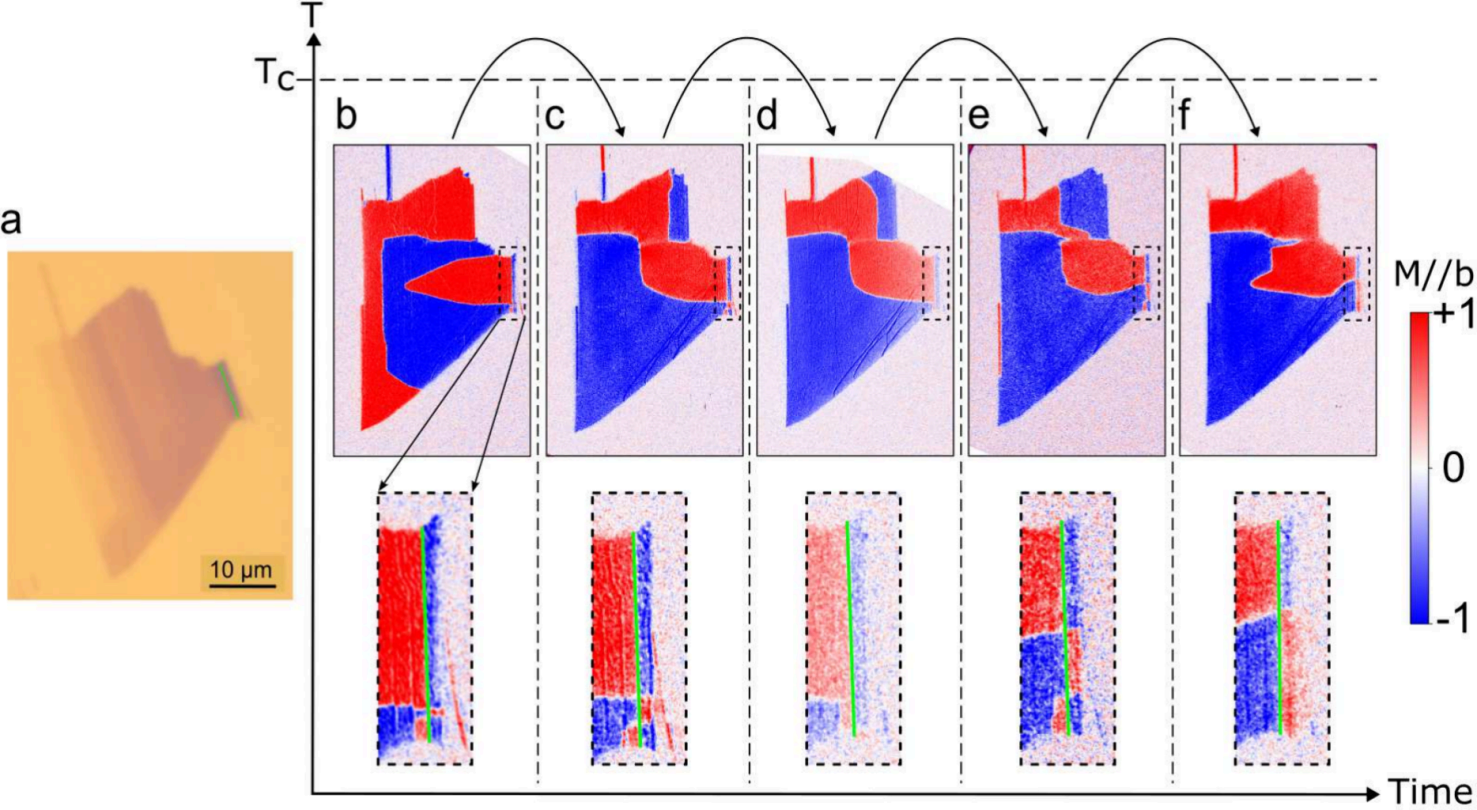
Magnetic domain patterns after warming-cooling cycles. **a** Optical microscopy image of the CrSBr flake on Au film shown in Figure 1. Lighter (darker) brown regions indicate thinner (thicker) parts of the CrSBr flake. The green line indicates the position of a topographic step on the 2D magnet surface. **b** SPLEEM image of the magnetic domain pattern in the entire CrSBr flake after the initial cooling down (same as in Figure 1e). **c**–**f** SPLEEM images reporting the formed domain patterns after each thermal cycle (warming-up above *T*_C_ and cooling down below *T*_C_). A ferromagnetic state in the thin CrSBr flake is formed each time *T* < *T*_C_. The bottom raw shows a zoom-in on the thickest part of the flake (*t*_CrSBr_ > 11 nm) where an antiferromagnetic interlayer coupling is observed.

Importantly, the SPLEEM images at the bottom of Figure 3 show a zoom-in on the thickest part of the flake (*t*_CrSBr_ > 11 nm), where we observe that the expected interlayer antiferromagnetic coupling of free-standing CrSBr is re-established. The green line indicates the position of the topographic step, where *t*_CrSBr_ increases from the left to the right.

To explore the potential origins of the emergent ferromagnetic interlayer exchange coupling in the CrSBr/Au system, we employ spatially resolved reflected electron spectroscopy (RES), also known as LEEM-*I*(*V*) spectroscopy.^26^ The aim is to better understand the role played by the interface between the semiconducting CrSBr flake and the metallic Au thin film in the emergent ferromagnetic ground state. RES spectra are qualitatively an inverted image of the unoccupied density of states (DOS)—above the vacuum level—of the sample, and in particular they allow us to determine the vacuum level (*E*_vac_) position. Figure 4a presents the RES spectra acquired on the CrSBr flake on Au (red dashed-dotted curve), as well as on the Au bare surface (black dashed curve). The distinct reflectivity drop in the RES spectra indicates the energy threshold above which the electrons can penetrate the surface of each material, that is, *E*_vac_, while the features at higher sample biases are linked to the unoccupied DOS at the sample surface. Two main differences can be identified between the RES spectra acquired on CrSBr and those on Au. First, the RES spectrum acquired on the CrSBr flake has a drop-off at a larger sample bias. Second, the RES spectrum acquired on the CrSBr flake has distinctive features for sample biases above the vacuum level, while the RES spectrum from the Au film is featureless. The former observation allows us to state that *E*_vac_ of the CrSBr flake is approximately 0.4 eV higher than that of the Au thin film, while the latter is proof of the single-crystal nature of the CrSBr flake and of the polycrystalline nature of the Au thin film.^26^

**Figure 4:**
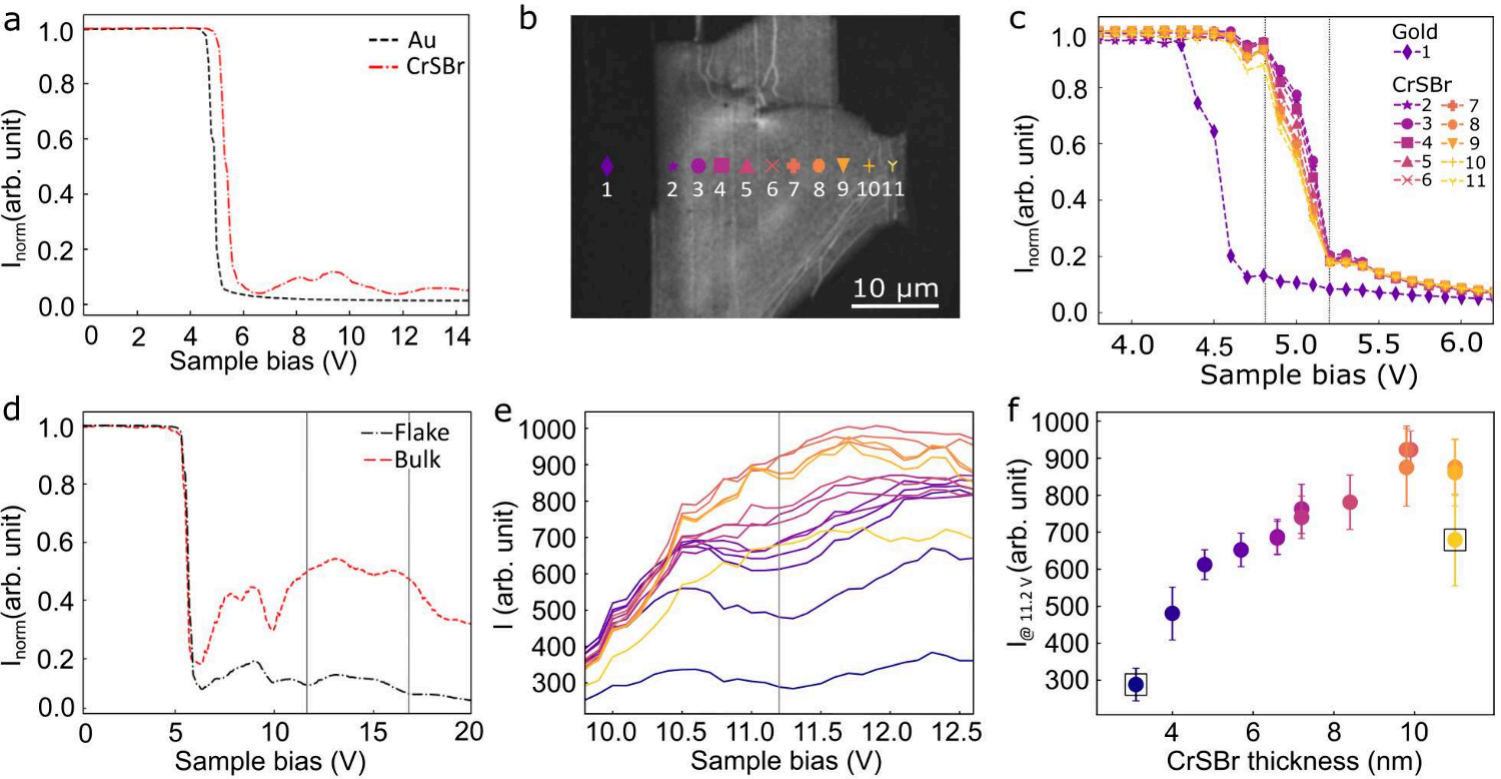
Reflected-electron spectroscopy on the CrSBr­(*t*_CrSBr_)/Au system. **a** RES spectra showing the reflected electron beam intensity as a function of the sample bias at the polycrystalline gold substrate (gray dashed curve) and at the center of the CrSBr flake (red dotted-dashed curve). **b** Locations on the sample where the RES spectra shown in panel **c** are acquired. Two dotted vertical lines in panel **c** indicate the transition between total electron reflectivity, *V* < 4.8 V and strong electron absorption, *V* > 5.2 V on the CrSBr flake. **d** RES spectra for bulk CrSBr (thickness ≫ 10 nm; red dashed curve) and the thin part of the flake of CrSBr (average over 3.5 < *t*_CrSBr_ < 8 nm; black dotted-dashed curve). The gray vertical lines indicate minima in the RES spectrum for the thin CrSBr that are not present in the spectrum of bulk CrSBr. **f** RES spectra acquired on the CrSBr flake at different thicknesses, going from the left edge (dark blue curve) to the right edge (yellow curve) of the flake shown in panel **b**. The gray vertical line indicates bias voltage of 11.2 V. **f** Reflected-electron intensity at a sample bias of 11.2 V, for different CrSBr thicknesses, extracted from RES spectra in panel **e**. Data points taken closest to the sample edges are highlighted with a black square. Error bars indicate the RMSQ of the RES intensity distributions and of the atomic force microscopy images corrugation. The error bars for the measured thickness are smaller than the symbols.

Next, we extract RES spectra across the CrSBr flake, going from the thinner (left) to the thicker (right) side of the CrSBr flake, as well as on the Au film, at the locations indicated on the LEEM image in Figure 4b. As the CrSBr thickness increases from approximately 3.5 to 11 nm toward the right side of the flake, any changes in the *I*(*V*) characteristic between these regions can be related to the CrSBr thickness. Figure 4c shows these RES spectra, zoomed in around the drop-off region. Here, the purple diamond curve indicates the spectrum of the Au layer, whereas the other curves indicate spectra taken on CrSBr of increasing thickness. The spectra taken on CrSBr all show similar behavior. The reflected intensity starts dropping quickly around a bias voltage of 4.8 V (see vertical dotted line on the left), after which it flattens out at a bias voltage of 5.2 V (see vertical dotted line on the right). Accordingly, within the energy resolution of the instrument (e-beam energy spread ≈ 200 meV^27^), we do not observe a clear shift of the vacuum level of CrSBr as a function of its thickness, for 3.5 ≤ *t*_CrSBr_ ≤ 11 nm.

The difference between the extracted *E*_vac_ on CrSBr and Au indicates the presence of a built-in potential, *V*_bi_, at the interface. *V*_bi_ results in the redistribution of charges between Au and CrSBr, forming an electron accumulation region in the semiconductor. By solving Poisson’s equation within a full depletion approximation, the dopant density can be expressed in terms of *V*_bi_ and the width of the accumulation layer *W* as $N_{D}=\frac{2\epsilon_{0}\epsilon_{r}V_{bi}}{qW^{2}}$ . Here ϵ_0_ is the vacuum permittivity, ϵ_r_ is the relative permittivity of CrSBr, *q* is the dopant concentration. This expression assumes the CrSBr and Au are undoped in the bulk. Though some doping may be present from the material growth and/or the device fabrication process, this doping is likely small. As no thickness-dependent band-bending could be directly observed (see Figure 4c), an upper limit of 3.5 nm is taken for *W*. Using a value of 10 for ϵ_r_ as reported by Ruta and colleagues,^28^ this yields a minimum electron doping density of *N*_D_ ≈ 10^19^ cm^–3^, corresponding to a sheet density of about 10^13^ cm^–2^.

The unoccupied DOS of the CrSBr flake is further investigated in Figure 4d, where the RES spectrum is plotted for two different thicknesses of CrSBr. The black dashed-dotted curve shows the average spectrum taken on the thin part of the CrSBr flake in Figure 4b (3.5 < *t*_CrSBr_ < 8 nm), whereas the red dashed line corresponds to a bulk CrSBr sample (*t*_CrSBr_ ≫ 10 nm). Above *E*_vac_, both curves show several features corresponding to the unoccupied DOS of the material. Minima (high DOS) and maxima (low DOS) in the two curves occur at different sample biases, indicating that the electronic structure above the vacuum level is different for the bulk and the thin flake. In particular, two minima at bias voltages equal to 11.2 and 16.4 V (see vertical gray lines in Figure 4d) present in the RES spectrum acquired on thin CrSBr on Au are not present in the spectrum acquired on the bulk CrSBr crystal. The spectral feature at sample bias = 11.2 V is further investigated in terms of the CrSBr thickness. Figure 4e shows 14 RES spectra acquired across the CrSBr flake shown in Figure 4b from the left edge to the right edge, in the 9.8–12.6 V bias voltage range. A clear thickness dependency in the *I*(*V*) spectra can be observed, where the minimum at sample bias = 11.2 V visible for the thinnest part (blue curves) of the CrSBr flake is not observed for the thicker part (orange-yellow curves) of the flake, transitioning toward a more bulk-like character (see dashed red curve in Figure 4d). Finally, Figure 4f shows the reflected-electron intensity at sample bias = 11.2 V as a function of *t*_CrSBr_, further highlighting the evolution of such spectral feature in the thickness range of the investigated CrSBr flake. It is worth noting that the two data points in Figure 4f coming from locations on the CrSBr surface in proximity to the edges show a strongly reduced RES intensity (see data points enclosed in black squares). This is understood as a measurement artifact due to the semiconducting nature of the 2D magnet, which results in the distortion of the electric field lines at the sample’s edges. Nonetheless, the rest of the data points in Figure 4f show an evident thickness dependency. This is experimental evidence of thickness-dependent band renormalization in the thin CrSBr flake,^17^ which can potentially originate from interface effects such as band hybridization^29^ and/or screening.

To investigate the potential origin of the observed ferromagnetic interlayer exchange interaction in the thin CrSBr flakes in proximity to a metallic film, we perform first-principles density functional theory (DFT) calculations. Strong electron doping has been previously observed in a few-layer CrSBr on a gold substrate.^29^ As a similar sample structure is employed here, electron doping is expected to be present. To investigate the influence of such doping on the magnetic ground state of the system, we first calculate for bulk structures the difference of the total energies of the ferro- and antiferromagnetic interlayer ground states, denoted by *E*_FM_ and *E*_AFM_, respectively, as a function of electron and hole doping. We add the latter in reference to gate-induced charge transfer that can also span into the hole doping regime. As shown in Figure 5a, at zero doping the interlayer antiferromagnetic state is energetically slightly more favorable (in our calculations by about 0.2 meV/Cr), in qualitative agreement with previous calculations^11,30,31^ and as expected for free-standing CrSBr.^22^ However, both electron and hole doping are found to promote the emergence of a ferromagnetic interlayer coupling over the antiferromagnetic alignment in bulk CrSBr. We note that Xie et al.^11^ found qualitatively the same trends in ab initio calculations for doped CrSBr bilayer.

**Figure 5:**
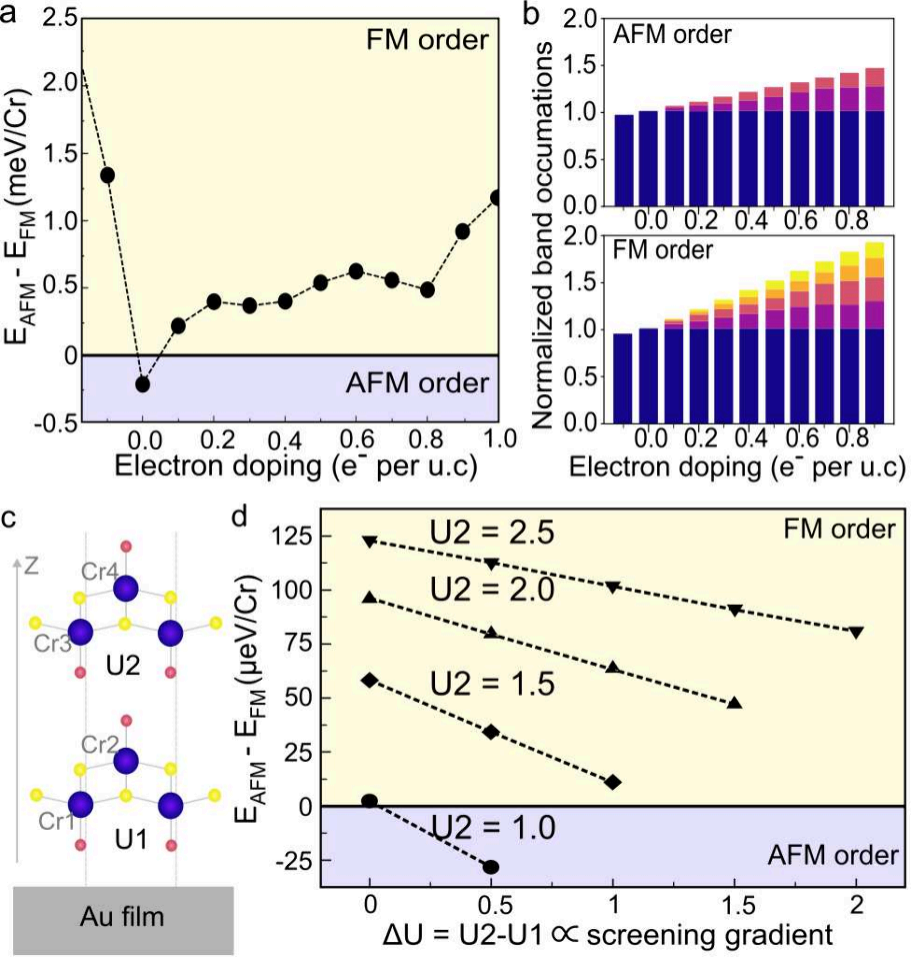
Computed dependence of interlayer exchange coupling in CrSBr with respect to electron doping and substrate screening. **a** The computed total energy difference between the antiferromagnetic (AFM) and the ferromagnetic (FM) stacking in bulk CrSBr. Both positive and negative electron doping are found to promote the emergence of an interlayer FM alignment over the AFM one expected for a free-standing CrSBr flake. **b** The band occupation normalized to the valence band occupation at zero doping as a function of the electron doping for both a fixed AFM (top) and FM (bottom) ordering, respectively. The blue bars refer to the valence band occupation, whereas the purple, red, orange and yellow bars correspond to different conduction bands. **c** Schematic of a CrSBr bilayer with different local Coulomb interaction, *U*, for top and bottom layer used to explore the effect of electrostatic screening from the Au metallic layer on the interlayer exchange interaction. **d** The computed total energy difference between the AFM and the FM stacking in bilayer CrSBr as a function of *U*_2_-*U*_1_. A gradient in *U* is always found to promote an AFM ordering against a FM one, while a large *U* promotes a FM ordering.

Even though the ferromagnetic arrangement is observed to become more and more energetically favorable for increasing electron doping, the trend is not entirely continuous. This results from the subsequent occupation of the conduction bands, which is different in the AFM and FM order. As shown in Figure 5b, in the case of fixed antiferromagnetic interlayer coupling, the doping electrons occupy only two conduction bands (purple and red bars), while, in the fixed ferromagnetic state, there are up to four conduction bands that get partially occupied (purple, red, orange, and yellow bands). On the hole doping side, we find the FM state energetically much more favored, with an (initially) more homogeneous trend in *E*_AFM_ – *E*_FM_ which is accompanied by a similar depopulation of the same highest valence bands in both interlayer coupling calculations.

The outcome of the first-principles DFT calculations speaks for a possible role played by the Fermi surface in the definition of the magnetic properties of CrSBr flakes with electron/hole doping. One possible scenario which could explain the asymmetric response to the type of doping, is that of a carrier-mediated Ruderman–Kittel–Kasuya–Yosida (RKKY) interaction, favoring the FM state. Since the Fermi surfaces at hole and electron doping are rather different,^32^ the RKKY interaction is expected to be rather different in the two cases, as it depends on both the effective mass and the Fermi wave vector. A second possible scenario is that of a superexchange mechanism between the neighboring layers being affected by electron/hole doping, as it results from virtual hopping from occupied to unoccupied sites. Thus, if the occupation of certain states changes upon doping, some virtual hoppings might be suppressed, affecting the superexchange mechanism.

A second aspect which is considered as a potential drive for the observed ferromagnetic ground state is screening. Indeed, having a thin layer of CrSBr in direct proximity to a metallic Au layer naturally raises the question of whether the Coulomb interactions between the charge carriers in the thin magnetic semiconductor are strongly affected by the presence of a metallic layer underneath. For the discussion of interlayer magnetic coupling, we specifically investigate here how layer-dependent Coulomb interactions (changing in *z*-direction from layer to layer, perpendicular to the CrSBr-Au interface) might affect the interlayer magnetic coupling. This kind of gradient in the local Coulomb interactions, i.e., a change of *U* from layer to layer, is to be expected, as the screening from the Au layer decreases with an increasing distance from the CrSBr-Au interface. Accordingly, we explore the effect of such a gradient in the Coulomb potential, Δ*U* = *U*_2_ – *U*_1_, between the layers of bilayer CrSBr (see schematic in Figure 5c), with *U*_1_(≤*U*_2_) being the effective Coulomb repulsion in the CrSBr layer supposedly closer to the Au substrate, while *U*_2_ is the local Coulomb interaction in the second layer further away from the substrate. The results of the calculations reported in Figure 4d show that increasing Δ*U* has a tendency toward stabilizing an AFM coupling, as Δ*E*(Δ*U*) = *E*_AFM_ – *E*_FM_ decreases with Δ*U*, while large values of *U* favor a FM interlayer coupling.

The two analyses above show that Δ*E* is much more sensitive to the electron doping than to Δ*U*. Based on this comparison between electron doping and screening (gradient) trends, we can conclude that electron doping is expected to be one of the possible drives behind the emergence of the observed ferromagnetic interlayer coupling.

Our experimental analysis shows that individual layers in thin CrSBr flakes on Au couple ferromagnetically with each other. This is accompanied by the observation of band renormalization. On the theoretical side, it was found before that the electronic band structure of CrSBr changes with the magnetic interlayer order.^9,12,33,34^ Here we complement this by ab initio calculations showing that the stability of the interlayer antiferromagnetic coupling in pristine CrSBr is weak and that small perturbations, e.g., doping due to electron/hole transfer, can easily tip the system toward a ferromagnetic ground-state. Our detailed analysis furthermore showed that a gradient in the local Coulomb interactions between neighboring layers, as it might be induced by screening from the metallic Au film, has a (mild) tendency toward stabilizing an AFM coupling. As no thickness dependency is observed for the vacuum level, *E*_vac_, of the thin CrSBr flake, within the ≈200 meV experimental resolution, the expected electron doping from the Au substrate must be localized near the CrSBr-Au interface, with a lower bound for the doping concentration estimated to be on the order of *N* ≈ 10^19^ cm^–3^, or approximately 0.1 e^–^/u.c. This doping concentration is shown to promote ferromagnetic coupling in the CrSBr in our calculations. It is worth stressing that the AFM order in CrSBr is expected to be weak, in agreement with previous estimates of small interlayer magnetic couplings for pristine CrSBr^35^ and so prone to be influenced by external stimuli, as demonstrated by the observed strong responses to rather weak magnetic fields,^33^ gating,^34,36^ or stacking.^37^

A potential additional force behind the stabilization of the observed ferromagnetic phase is strain generated in the CrSBr flake by local bending during the exfoliation process. It was reported that tuning of the in-plane lattice constant in CrSBr could realize a ferromagnetic phase.^38^ However, we do not have any experimental evidence that connects the observed ferromagnetic phase with strain in our study. The strain induced in the exfoliated CrSBr is rather non-homogeneous, most likely varying locally from tensile to compressive across the flake and with no specific periodicity, in contrast with the uniform unidirectional strain considered in the study by Cenker et al.^38^ All of this seems to rule out strain as the main cause of the observed ferromagnetic phase in our system.

In conclusion, given the fact that charge transfer into the CrSBr flake is the most likely origin of the observed ferromagnetic phase, our findings suggest that the behavior of CrSBr-based devices with direct contact to metallic leads needs to be very carefully interpreted in terms of their interlayer magnetic coupling properties in the vicinity of the contacts. Finally, our study highlights the utility of directly imaging the magnetic ground state of thin van der Waals materials via SPLEEM and sheds new light on the possibility of engineering the magnetic properties of 2D van der Waals magnets via proximity/interface effects with metallic layers.

## Supplementary Material


