# Supplementary material for: Ferromagnetic Interlayer Exchange Coupling in a Few Layers of CrSBr on a Gold Thin Film
Source: Nano Lett. 2026 Aug 3;26(31):10164–71. doi: 10.1021/acs.nanolett.6c01730 (PMC13475040; doi:10.1021/acs.nanolett.6c01730)
Supplement: Supplementary file 1 [file nl6c01730_si_001.pdf]

Supplementary information for the manuscript "Ferromagnetic interlayer exchange coupling in a few layers of CrSBr on a gold thin film"

Rixt Bosma*,[†] Darius A. Pacurar*,[†] Daniel Sade,[†] Jingbo Wang,[†] Nicholas Dale,[‡,§] Cameron W. Johnson,[‡] Sergii Grytsiuk,[¶] Alexander Rudenko,[¶] Alexander Stibor,[‡] Malte Rösner,[¶,‖] Marcos H. D. Guimarães,[†] and Roberto Lo Conte[*,†]

†*Zernike Institute for Advanced Materials, University of Groningen, Groningen, The Netherlands*

‡*Molecular Foundry, Lawrence Berkeley National Laboratory, Berkeley, California, USA*

¶*Institute for Molecules and Materials, Radboud University, Nijmegen, Netherlands*

§*Materials Science Division, Lawrence Berkeley National Laboratory, Berkeley, California, USA*

‖*Faculty of Physics, Bielefeld University, Bielefeld, Germany*

E-mail: r.lo.conte@rug.nl

Section S1. Sample fabrication

SiO_x/Si substrates are cleaned in acetone. 5 nm of titanium and 20 nm of gold is deposited onto the substrate via electron beam evaporation. Bulk CrSBr crystals were purchased from HQ graphene and exfoliated in a nitrogen-filled glove-box using mechanical exfoliation onto a commercial PDMS film (gel pak). Suitable flakes were selected based on optical contrast and transferred onto the gold-coated SiO_x/Si substrate.

Section S2. Low-temperature magnetic imaging via SPLEEM

The magnetic imaging of the CrSBr flakes on Au/SiO_x/Si substrates, as well as the reflected-electron spectroscopy measurements, were conducted with the low-temperature SPLEEM system (Elmitec Model LEEM III with spin-polarized electron beam source, spin-manipulator and custom-designed liquid helium cryostage) available at the Molecular Foundry of the Lawrence Berkeley National Laboratory. The system allows for the acquisition of images via low-energy electrons with magnetic contrast by employing a spin-polarized electron beam as the illumination source. The spin orientation of the electron beam can be selected along any desired direction in three-dimensional space. The SPLEEM images were acquired with samples cooled down via a liquid-He flow cryostat. The magnetic contrast is a direct result of the different reflection probability for spin-up and spin-down electrons at the magnetic surface. The origin of such spin-dependent electron reflection probability is the spin-polarized density of states of the magnetic surface. The magnetic asymmetry in a SPLEEM image is calculated as $A = [(S_u - S_d)/(S_u + S_d)]$, where S_u and S_d are the backscattering intensity of the spin-up and spin-down polarized electron-beam, respectively. Images are drift-corrected before performing averaging in order to maximize the quality of the presented SPLEEM images. The drift correction is performed via cross-correlating all the LEEM images contained in the acquired image stack.

Section S3. Further observations of ferromagnetic interlayer coupling in thin CrSBr flakes on Au thin films

In Fig. S1 we report the observed magnetic domain pattern hosted by another thin CrSBr flake transferred onto a Au thin film. For the preparation of this sample, we used a different CrSBr crystal and a different Au/SiO_x/Si substrate than the ones used to make the sample discussed in the main manuscript. Fig. S1a shows an atomic force microscopy (AFM) image of the CrSBr flake, while Fig. S1b presents the magnetic domains observed via low-temperature SPLEEM. The black segments indicate the paths along which three line profiles were extracted from the AFM image in Fig. S1a. Figure S1c-e report the actual line profiles, showing the height profile of the CrSBr surface at those specific locations. Notably, several CrSBr monolayer steps can be identifies (see double-arrowed red segments). All those single layer steps are located inside a magnetic single domain, demonstrating the presence of a ferromagnetic interlayer coupling.

Section S4. Reflected-electron spectroscopy

During the acquisition of reflected-electron spectra, the landing energy of the electrons is controlled via the application of a bias voltage (starting voltage) between sample and cathode. Reflected-electron spectroscopy is conducted by varying the landing energy of the electrons from a low value to a high value. In the low energy regime, the electrons energy is lower than the vacuum level of the sample and are fully reflected (mirror mode). At a particular applied bias voltage, the electrons have enough energy to penetrate the sample surface and probe the unoccupied energy levels above the vacuum level at the sample's surface (LEEM mode). A custom-developed Python code was employed to extract spatially resolved reflected-electron spectra from the acquired LEEM-$I(V)$ image stacks.

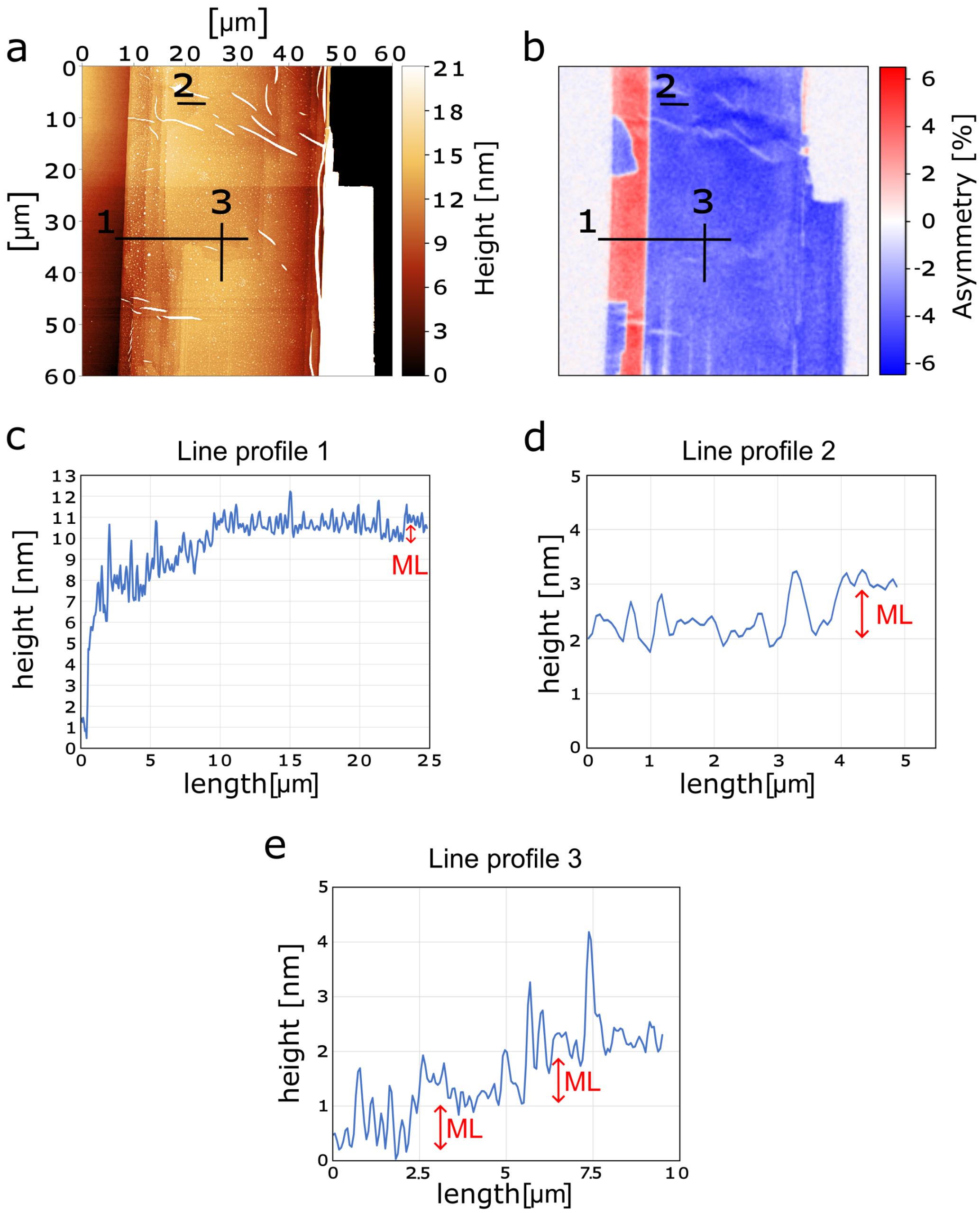

Figure S1: Structural and magnetic characterization of one of the investigated CrSBr-flake/Au(20 nm)-thin film sample. **a** Atomic force microscopy image of CrSBr flake on Au thin film on SiOx/Si wafer. The black segments indicate the location where three line profiles were acquired, named as 1, 2 and 3. **b** SPLEEM image of the same CrSBr flake in a, showing the magnetic domain pattern hosted by the magnet. **c-e** Three line profiles showing the corrugation at the CrSBr surface at the location of the three black segments on the AFM image in panel a. In particular, monolayer steps are indicated by red double-arrowed segments. From the comparison among the AFM image, the SPLEEM image and the line profiles, it clearly appears that there is no magnetic contrast switching at monolayer steps.

Section S5. DFT calculations

All DFT calculations are performed within the projected augmented wave formalism[1] as implemented in the Vienna Ab-Initio Simulation Package[2,3] (VASP). The exchange-correlation effects were considered within the GGA-PBE functional[4]. To account for the on-site Coulomb repulsion within the $3d$ shell of Cr atoms, we used the standard DFT+U scheme[5] with $U = 4\,\text{eV}$ and $J = 1\,\text{eV}$. To ensure sufficient numerical accuracy of our results, we apply a $(16\times12\times4)$ k-point grid for Brillouin zone integrations, and an energy cut-off of 800 eV for the plane waves. The lattice constants and atomic positions are taken from the Materials Project for orthorhombic CrSBr (mp-22998), and then fully relaxed using the parameters of our calculations at charge neutrality, yielding $a \approx 3.45\,\text{Å}$, $b \approx 4.66\,\text{Å}$ and $c \approx 15.56\,\text{Å}$. To capture the van der Waals (vdW) dispersion forces between the CrSBr layers during the relaxation, we apply a vdW correction within the DFT-D3 method[6]. We note that to stabilize the interlayer AFM ground state at charge neutrality both, applying $J = 1\,\text{eV}$ and the full relaxations were important. Thus, the lattice structure and the applied Coulomb parameters differ slightly from previously used ones[7].

References

(1) Kresse, G.; Joubert, D. From ultrasoft pseudopotentials to the projector augmented-wave method. *Phys. Rev. B* **1999**, *59*, 1758–1775.

(2) Kresse, G.; Hafner, J. Ab initio molecular dynamics for liquid metals. *Physical Review B* **1993**, *47*, 558–561.

(3) Kresse, G.; Furthmüller, J. Efficient iterative schemes for ab initio total-energy calculations using a plane-wave basis set. *Physical Review B* **1996**, *54*, 11169–11186.

(4) Perdew, J. P.; Burke, K.; Ernzerhof, M. Generalized Gradient Approximation Made Simple. *Phys. Rev. Lett.* **1996**, *77*, 3865–3868.

(5) Liechtenstein, A. I.; Anisimov, V. I.; Zaanen, J. Density-functional theory and strong interactions: Orbital ordering in Mott-Hubbard insulators. *Phys. Rev. B* **1995**, *52*, R5467–R5470.

(6) Grimme, S.; Ehrlich, S.; Goerigk, L. Effect of the damping function in dispersion corrected density functional theory. *Journal of Computational Chemistry* **2011**, *32*, 1456–1465.

(7) Rudenko, A. N.; Rösner, M.; Katsnelson, M. I. Dielectric tunability of magnetic properties in orthorhombic ferromagnetic monolayer CrSBr. *npj Computational Materials* **2023**, *9*, 83.
